# Emergency diagnosis and management of pediatric arrhythmias

**DOI:** 10.4103/0974-2700.66525

**Published:** 2010

**Authors:** Carla R Hanash, Jane E Crosson

**Affiliations:** The Johns Hopkins University School of Medicine, Department of Pediatrics, Division of Pediatric Cardiology, 600 North Wolfe Street, Baltimore, MD 21287-2651

**Keywords:** Congenital heart disease, cardiomyopathies, narrow-complex tachycardia, pediatric arrhythmias, supraventricular tachycardia, wide-complex tachycardia

## Abstract

True emergencies due to unstable arrhythmias in children are rare, as most rhythm disturbances in this age group are well-tolerated. However, presentation to an emergency department with symptoms of palpitations, fatigue and/or syncope is much more common. Sinus tachycardia is by far the most commonly reported arrhythmia, followed by supraventricular tachycardia. Emergency physicians should be prepared for diagnosis and to acutely manage various types of arrhythmias seen in children, to assess the need for further diagnostic testing, and to determine whether cardiology evaluation and follow-up are needed. This article is intended to provide diagnostic and management guidelines of the most common types of arrhythmias seen in children with structurally normal hearts as well as those associated with congenital heart disease and cardiomyopathies.

## INTRODUCTION

**Figure 1:**
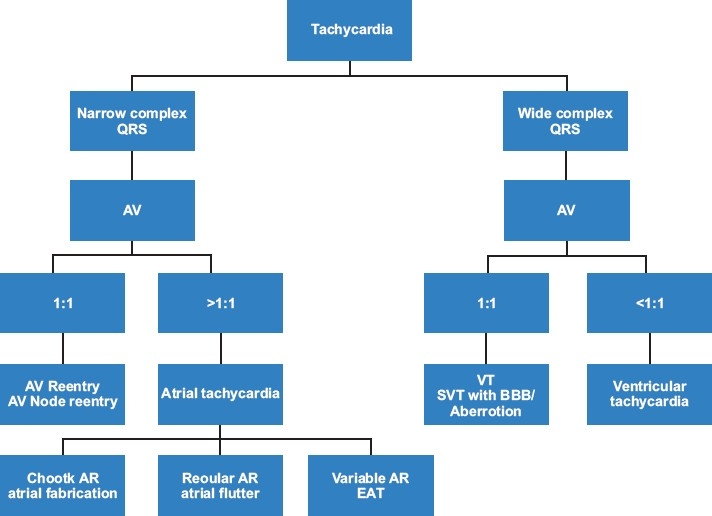
Diagnostic algorithm to determine underlying mechanism of various tachycardias. AV, atrioventricularratio; SVT, supraventricular tachycardia; BBB, bundle brunch block; AR, atrial rate; EAT, ectopic atrialtachycardia; VT, ventricular tachycardia

True emergencies due to unstable arrhythmias in children are rare, as most rhythm disturbances in this age group are well-tolerated. However, presentation to an emergency department with symptoms of palpitations, fatigue and/or syncope is much more common. Pediatric arrhythmias account for approximately 55.1 per 100,000 patients evaluated in pediatric emergency departments. Sinus tachycardia is by far the most commonly reported arrhythmia, followed by supraventricular tachycardia (SVT) which represents about 13%, and bradycardia accounting for about 6% of all cases.[1] Emergency physicians should be prepared for diagnosis and to acutely manage various types of arrhythmias seen in children, to assess the need for further diagnostic testing, and to determine whether cardiology evaluation and follow-up are needed. This article is intended to provide diagnostic [Figure 1] and management guidelines of the most common types of arrhythmias seen in children with structurally normal hearts as well as those associated with congenital heart disease and cardiomyopathies.

## TACHYCARDIAS

### Narrow QRS complex tachycardia (supraventricular tachycardia)

**Figure 2:**
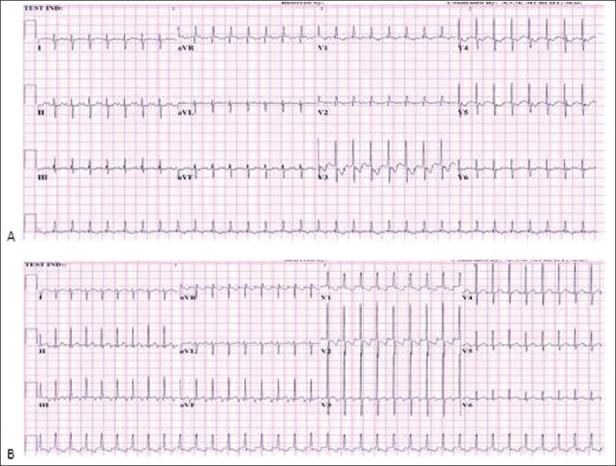
(a) Sinus tachycardia with normal P wave morphology; (b) SVT with abnormal P wave axis. Both ECGsare from neonates. Note the similar ventricular rates

Narrow complex tachycardia refers to any arrhythmia that originates above or at the bundle of His. These are by far the most common tachycardias seen in children, with an estimated incidence of 0.1–0.4%.[2] Three mechanisms of tachycardia have been described. In the most common, re-entry tachycardia, the circuit can originate within the atrium producing atrial flutter or fibrillation, at the level of the AV node (AV node reentry tachycardia), or via an accessory pathway [AV re-entry tachycardia (AVRT)]. A second mechanism is automatic tachycardia, which results from an enhanced automatic focus. This can be within the sinus node as in sinus tachycardia or in foci elsewhere in the atria. Sinus tachycardia is commonly seen in the setting of fever, anemia, hypovolemia, and medications known to increase catecholamines and is uncommonly associated with underlying cardiac disease. It can be distinguished from SVT by the presence of a normal sinus P wave preceding every QRS complex [Figure 2a]. Sinus tachycardia rates can exceed 140 bpm in children and 180 bpm in infants but are usually less than 200 bpm. Treatment is primarily aimed at the underlying disorder. Triggered tachycardia is a much less common tachycardia mechanism except in the setting of drug toxicity such as digoxin overdose.

### A) AV reentry and AVN reentry tachycardia

### 1. AVRT

**Figure 3:**
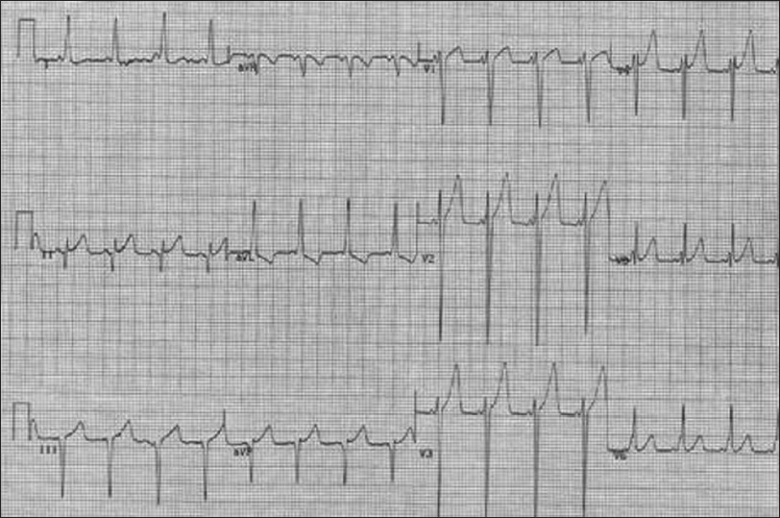
WPW syndrome. Note the short PR interval and slurred QRS upstroke (delta wave)

This is also known as accessory-pathway mediated tachycardia or orthodromic reciprocating tachycardia, and is by far the most common type of SVT seen in children, representing 82% of arrhythmias occurring during infancy.[3] AVRT involves two distinct pathways between the atria and ventricles, which create an electrical reentry circuit proceeding down the AV node and then up an accessory pathway outside the AV node creating a narrow QRS complex tachycardia [Figure 2b]. The much rarer *antidromic* tachycardia reverses the direction of conduction, with transmission down the accessory pathway and up the AV node, creating a widened QRS complex, which will be discussed under wide complex tachycardias. Once normal sinus rhythm is re-established, 50% of patients manifest ventricular pre-excitation in the form of a delta wave on electrocardiogram, consistent with Wolf-Parkinson-White syndrome (WPW); the remainder of patients have a “concealed” pathway that is not evident during sinus rhythm. The only difference between the two forms is the presence of manifest antegrade conduction to the ventricle in WPW. Because conduction through the pathway can occur before the AV node is activated, a shortened PR interval slurred QRS upstroke and widensed QRS result [Figure 3]. This also creates the potential for rapid ventricular response during atrial fibrillation, which can result in ventricular fibrillation and sudden death. Thus, although acute management of SVT is the same for patients with concealed and manifest pathways, definitive ablative therapy in patients with WPW is compelling.

### 2. AV Node reentry tachycardia (AVNRT)

This is also is due to a re-entry circuit but with pathways that involve the AV node. In this tachycardia, there are also two discrete conduction limbs, a “slow” pathway which usually conducts antegrade, and a so-called “fast” pathway which usually conducts retrograde. This accounts for ~15% of SVT in the pediatric age group, increases with age, and is rarely seen in infants.[4] These two types of tachycardia are often clinically indistinguishable, having similar rates and being well-tolerated in most patients. The exception is the infant with AVRT, in whom tachycardia can go unrecognized for prolonged periods, resulting in gradual cardiovascular deterioration. Fortunately, initial therapy is identical in both types of SVT. Briefly interrupting conduction through the AV node with adenosine terminates both types, and long-term medication or ablative choices are very similar.

#### Clinical features

- History of poor feeding, lethargy, irritability, pallor in infants;
- Historyof palpitation, dizziness, chest pain, syncope and shortness of breath in older children and adolescents;
- Signs and symptoms of congestive heart failure in some infants and
- History of abrupt onset and termination.

#### Electrocardiographic findings

- These are presented in Figure 2. The important findings are as follows:
- HeartRates >220 in infants and >180 in children;
- Narrow QRS complex and
- AV ratio 1:1
- In AVNRT, terminal QRS notching ± visible.
- In AVRT, inverted P waves ± visible.

#### Acute management

- Attempt vagal maneuver (i.e., application of ice, Valsalva maneuver or carotid massage);
- if unsuccessful, give adenosine 100 mcg/kg rapid bolus, increase the dose and repeat as needed;
- Synchronized DC cardioversion if adenosine is unsuccessful, starting at 0.5J/kg and
- Intravenous amiodarone alternative in experienced hands.

#### Further work-up and disposition

- Once normal sinus rhythm is established, repeat electrocardiogram to rule out pre-excitation and underlying cardiac disease.
- Laboratory work-up: serum electrolytes, thyroid function tests and complete blood count.
- All patients under 1 year of age and those with hemodynamically unstable SVT should be admitted for observation. Patients with first time episode and normal hearts should be observed overnight, without treatment. In patients with known structural heart disease, therapy is recommended on an individualized basis. If patient is discharged, provide instruction on vagal maneuvers to treat recurrence, as well as a list of indications to seek medical attention.
- Initiation of maintenance therapy would depend on patient age and severity of symptoms and recurrence, and should be discussed with cardiologist.
- Cardiology follow-up is recommended for all new patients. Provide a copy of EKGs to the parents, to be brought during their visit.
- Infants with suspected prolonged episodes should have echocardiography to rule out thrombus and ventricular dysfunction. Otherwise, echocardiogram may be obtained during cardiology evaluation to rule out underlying heart disease.
- Of note, patients found to have asymptomatic pre-excitation on EKG should also be referred for cardiology evaluation.

#### Prognosis

The probability of complete resolution of SVT is dependent on age of onset. In the majority of infants diagnosed at 1 year of age or less, SVT is likely to resolve, compared to only 33% of patients diagnosed after 1 year of age.[5] Maintenance therapy includes the use of beta-blockers in cases of recurrent and/or prolonged tachycardia. Digoxin and calcium channel blockers should not be prescribed in patients with WPW prior to full cardiology evaluation. Sotalol, flecainide or amiodarone may be necessary for medical control of some SVT. Catheter ablation allows for definitive therapy for SVT, at low risk in patients beyond the toddler years.

### B) Atrial tachycardia

### 1. Atrial flutter

In children, atrial flutter usually occurs in fetal life or shortly following birth. It is rarely seen beyond the newborn period except in the setting of underlying congenital heart disease and cardiac surgery, where it is most common in children who have undergone Fontan procedures, atrial septal closure and tetralogy of Fallot (TOF) repair. Congenital heart disease and post-operative patients represent about 80% of older children presenting with atrial flutter.[6] It accounts for about 30% of fetal tachycardia, 18% of tachycardias in newborn and only 8% in older children.[7] Atrial flutter involves a single reentry circuit within the atrial muscle, most commonly around the borders of the tricuspid valve. Hemodynamic compromise is determined by the duration of flutter and the degree of AV block, with 1:1 AV conduction resulting in the most significant instability. Clinical presentation depends on ventricular response. In the setting of rapid ventricular conduction, patients may be hemodynamically unstable with poor cardiac output.

#### Clinical features

- Diagnosed *in-utero* during fetal ultrasound evaluation;
- Fetal hydrops common if prolonged;
- Most newborns are asymptomatic unless tachycardic>48hours;
- Infants with prolonged tachycardia may present with history of poor feeding, irritability, lethargy, diaphoresis and pallor and
- Older children may complain of palpitations, chest pain and/or dizziness.

#### Electrocardiographic findings

**Figure 4:**
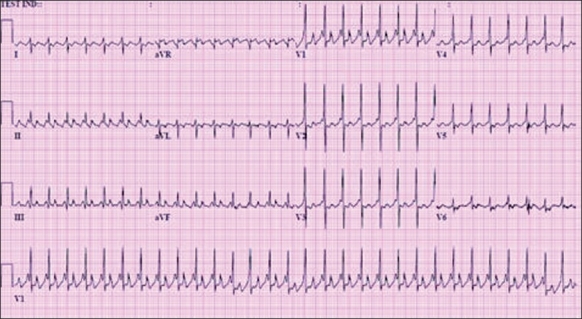
Atrial flutter in a newborn with atrial rate of 375 and ventricular rate of 185 bpm. Note the typical “sawtooth” appearance and AV conduction >1:1

The findings are presented in Figure 4. They are as follows.

- Regular atrial rates of 240–360 bpm;
- AV ratio >1:1, variable but generally regular, ventricular rate 120–240;
- Typical sawtooth appearance may be seen in lead II, III and aVF and
- Normal appearing QRS complex.

#### Acute management

- Vagal maneuvers or adenosine do not convert this rhythm but may increase the degree of AV block, unmasking underlying flutter waves.
- If patient has rapid ventricular response with reduced cardiac output, or for elective cardioversion, use DC cardioversion starting at 0.5J/kg, increasing to 1–2J/kg if needed.
- If stable, pharmacotherapy can be aimed at rhythm control with IV amiodarone or ibutilide, or at rate control with beta-blockers or calcium-channel blockers.
- In patients with pacemaker, pace termination can be attempted by the cardiologist.

#### Further work-up and disposition

- Laboratory work-up: serum electrolytes and thyroid function tests;
- Cardiology consultation and echocardiogram to rule underlying structural heart disease, tachycardia-mediated cardiomyopathy and thrombus;
- Admit for observation and
- Anticoagulation therapy for episodes >48 hours and in all Fontan patients.

#### Prognosis

In the presence of a structurally normal heart, the risk of recurrence of neonatal atrial flutter is low and no long-term therapy is needed. In cases of structurally abnormal heart and/or recurrent atrial flutter, long-term therapy may be required. Management is directed at either rhythm control with agents such as amiodarone and flecainide, or control of ventricular response with digoxin, calcium channel blockers or beta-blockers in selected patients. Catheter ablation can be highly successful in the older child.

### 2. Ectopic atrial tachycardia

Ectopic atrial tachycardia is uncommon, accounting for about 10% of SVT in children.[8] It is due to enhanced automacity of single or multiple foci outside the sinus node and is often refractory to medical therapy and cardioversion. Ectopic atrial tachycardia is the most common cause of tachycardia-induced cardiomyopathy due to its persistent and chronic nature; improvement in cardiac function has been observed following successful treatment. The precise etiology is unknown; however, viral illness, atrial tumors and genetic predisposition have been implicated. Clinical presentation depends on the extent of cardiac dysfunction, with most patients presenting with minor symptoms in the setting of preserved function.

#### Clinical features

- Is predominantly observed in infants and children with structurally normal hearts.
- Patients present with symptoms of chest pain, palpitations, pre-syncopal or syncopal events. Older children may present with exercise intolerance or be asymptomatic.
- Infants may present with feeding difficulties, diaphoresis with feeds or in respiratory distress secondary to chronic tachycardia.

#### Electrocardiogram findings

**Figure 5:**
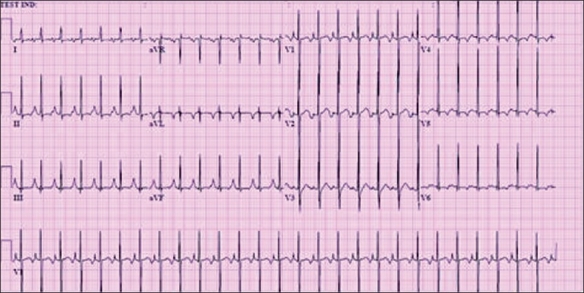
EAT in an asymptomatic 8-month patient with abnormal P wave axis. Note merging of T and P waves

These are shown in Figure 5. The findings are as follows:

- Atrial rate that is inappropriately rapid for age. Sometimes difficult to distinguish from sinus tachycardia;
- Abnormal P wave morphology and axis;
- Atrial rate variable, ranging from 120 to 300 bpm;
- May exhibit a “warm up” period at initiation with progressive P-P interval shortening and a “cooling down” period prior to termination and
- >1:1 AV conduction can be seen in patients at rest or while asleep without termination of the tachycardia.

#### Acute management

- Responds poorly to adenosine and DC cardioversion.
- First line treatment for symptomatic patients includes IV amiodarone. Load with 5mg/kg bolus over 20–60 minutes.
- Can be followed by amiodarone maintenance drip at 10–15mg/kg/day.
- Minimally symptomatic patients do not require acute treatment.

#### Further work-up and disposition

- Laboratory work-up: serum electrolytes, complete blood count, toxicology screen. If cardiac function is poor, additional laboratory evaluation should include viral panel, blood culture and cardiac enzymes to help differentiate infection versus tachycardia-induced cardiomyopathy.
- Cardiology consultation and echocardiographic evaluation is recommended.
- Obtain 24-hour cardiac monitor to determine duration of tachycardia and ventricular rates.
- Treatment is dependent on patient age, symptoms, clinical status, tachycardia rate and duration, and ventricular function.
- Asymptomatic patients can be treated conservatively.
- Children younger than 1 year and symptomatic patients should be admitted for management of arrhythmia and congestive heart failure. In the setting of normal cardiac function, beta-blockers can be useful by slowing AV node conduction, lowering the ventricular rate and improving symptoms.
- More aggressive treatment consists of flecainide, amiodarone or sotalol which have moderate success rates. Patients who fail medical therapy can undergo catheter ablation.

#### Prognosis

Spontaneous resolution may be observed in 30–50% of affected children.[9] Children younger than 3 years have higher incidence of AET resolution with treatment versus older children who are likely to require RF ablation because of persistent tachycardia despite optimal medical management.[10]

### 3. Atrial fibrillation

Atrial fibrillation is mostly seen in children with underlying structural heart disease and in those who have undergone cardiac surgery. In infants and children with structurally normal hearts, atrial fibrillation is often associated with an AV accessory connection such as WPW. These patients can be at increased risk of sudden death if there is rapid conduction to the ventricle via a manifest accessory pathway. Atrial fibrillation can also be associated with cardiomyopathies, myocarditis, pericarditis and hyperthyroidism, and in rare instances has a genetic predisposition. The mechanism involves multiple reentry circuits predominantly within the left atrium. Patients are usually symptomatic at presentation and in the setting of rapid ventricular rates, hypotension and syncope may ensue. As in adults, the possibility of atrial thrombus with the risk of embolic stroke is of great concern.

#### Clinical features

- Children and adolescents present with complaints of palpitations. Weakness and signs of congestive heart failure may be seen.
- In patients presenting with syncope, WPW should be highly suspected.

#### Electrocardiogram findings

**Figure 6:**
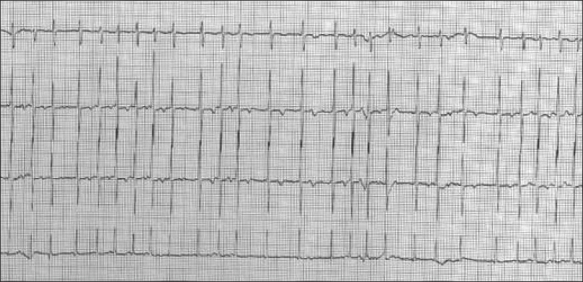
Atrial fibrillation in a 2½ year old asymptomatic patient. Note chaotic atrial waves with irregular ventricular response

Figure 6 shows the findings. They are as follows.

- Chaotic/irregular atrial waves best seen in lead V1;
- Atrial rates of 350–600 bpm;
- No discrete, uniform P waves;
- AV ratio >1:1;
- Variable, changing ventricular response rate (irregularly irregular), ranging from 110 to 200 bpm and
- Normal appearing QRS complex, except in WPW.

#### Acute management

- If the patient has rapid ventricular response with reduced cardiac output, DC cardioversion starting at 2J/kg is used.
- For stable patients, pharmacotherapy aimed at ventricular rhythm or rate control can be used. IV rhythm control agents include amiodarone and ibutilide. Less effective are sotalol, digoxin and procainimide.
- Cardioversion in stable patients with unknown or prolonged (>48 hours) duration of tachycardia should be delayed until assessment for atrial thrombus is made with echocardiography.

#### Further work-up and disposition

- Once in normal sinus rhythm, repeat EKG.
- Laboratory work-up: serum electrolytes, thyroid function, complete blood count, toxicology screen. If cardiomyopathy is suspected, additional laboratory evaluation should include viral panel, blood culture and cardiac enzymes.
- Echocardiography is indicated in all cases.
- Admit for observation and treatment.
- Initiate anticoagulation therapy in most cases.

#### Prognosis

Despite medical therapy, atrial fibrillation has a high recurrence rate and often requires catheter or surgical intervention. Chronic anticoagulation therapy is indicated in patients with persistent or recurrent atrial fibrillation. Ablation of the AV node with implantation of a pacemaker may be necessary in some refractory cases.

### Wide QRS complex tachycardias

Although less common in children than in adults, wide complex tachycardia still occurs with some frequency. As outlined in Figure 1, not all wide complex tachycardias are due to ventricular tachycardia (VT). The differential diagnosis of wide complex tachycardia includes VT, bundle branch block during SVT and SVT with pre-excitation in patients with WPW. If available, a previous 12-lead electrocardiogram may show underlying bundle branch block and can be helpful in distinguishing VT from SVT with aberrancy. Due to the potential life-threatening nature of VT, all wide complex tachycardia should be treated as VT until proven otherwise.

### A) Ventricular tachycardia

VT is a potentially life-threatening arrhythmia recognized as a cause of sudden death in both adults and pediatrics. It is rare in children and accounts for about 6% of patients followed for tachycardias.[11] Defined as a tachycardia originating below the bundle of His, rates can range from just over the sinus rate to well over 200 bpm. Episodes lasting less than 30 seconds are termed as non-sustained VT and those more than 30 seconds as sustained VT. VT can further be classified as monomorphic, with a regular rate and a single QRS morphology, versus polymorphic, with variability in rate and QRS morphology. The same basic mechanisms of automacity, reentry and triggered tachycardia exist in VT as for other arrhythmias. Etiology is widely variable and includes idiopathic, drug toxicity, cardiomyopathy, myocarditis, cardiac tumors and metabolic abnormalities, to name a few.

#### Clinical features

- Variable, may present with symptoms ranging from dizziness and palpitations to syncope and cardiac arrest.

#### Electrocardiogram findings

**Figure 7:**
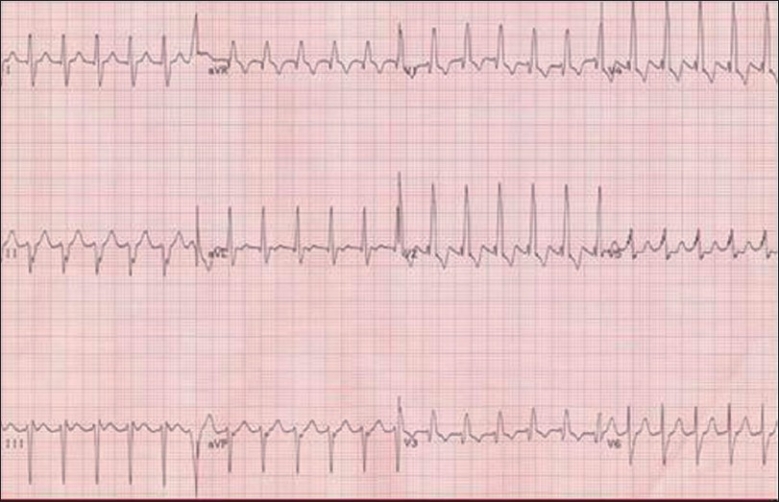
VT in patient with TOF

Figure 7 shows the findings which are as follows.

- Hallmarks are prolonged QRS duration for age and VA dissociation with ventricular rates exceeding atrial rates (AV ratio < 1:1).
- Rates range from ~110 to >200 bpm.
- When VA conduction is 1:1, VT cannot be excluded. Features consistent with the diagnosis of VT rather than SVT with a wide QRS include variation in RR interval and presence of fusion complexes.
- Complexes may appear uniform or vary from beat to beat as in polymorphic VT.

#### Acute management

- If unstable, synchronized cardioversionis started at 2J/kg and repeated, increasing the dose if needed.
- If stable, may attempt amiodarone at 5mg/kg IV over 30–60 minutes or procainimide at 15mg/kg IV over 30–60 minutes.

#### Further work-up and disposition

- History should focus on prior symptoms, symptoms suggestive of myocarditis or long-standing cardiomyopathy, and the possibility of drug toxicity, as well as a thorough family history for known arrhythmias or history of sudden death.
- Once in normal sinus rhythm, repeat EKG to rule out underlying abnormalities including long QT, Brugada, arrhythmogenic right ventricular cardiomyopathy, structural heart disease, electrolyte abnormalities and ischemia.
- Laboratory work-up should include toxicology screen, serum electrolytes, complete blood count, viral panel, blood culture and cardiac enzymes.
- Cardiac consultation and echocardiographic evaluation are done to rule underlying structural heart disease, cardiomyopathy, cardiac tumors.
- Admit for observation.
- After cardioversion, return to sinus rhythm may be transient and continual infusion of amiodarone may be required.

#### Prognosis

In the setting of a structurally normal heart and stable, well-tolerated monomorphic VT, many minimally symptomatic patients can be followed closely without therapy. Such patients may benefit from B-blockers to reduce ectopy. Chronic treatment otherwise depends on rate, duration, symptoms, type of VT and the presence of genetic channelopathy such as long QT. Aggressive antiarrhythmic therapy usually in addition to implantable cardioverterdefribrillator (ICD) placement is required for life-threatening VT.

### B) Long QT syndrome

The congenital long QT syndrome is a genetic disorder of prolonged cardiac repolarization that may cause cardiac arrest and sudden death. Abnormalities in cardiac ion channels predispose patients to a characteristic polymorphic VT called “torsades de pointes”. Events are often precipitated by adrenergic stimuli. Acquired long QT may also occur and can be caused by drugs, underlying medical conditions or electrolyte imbalances.

#### Clinical features

- Patients may present with presyncope, syncope, seizures, or cardiac arrest.
- Precipitating factors may include exercise, especially swimming, emotional stress, exposure to loud noises or even sleep.
- Although rare, infants can present with poor feeding, or with episodes of lethargy, cyanosis or poor perfusion.

#### Electrocardiogram findings

Sinus rhythm ECG, QTc of >460 in post-pubertal females and 450 in others, best obtained from lead II (Bazett Formula QTc= QT Interval/√-RR).

- Borderline QTc>440 ms in the setting of clinical symptoms and/or family history should be investigated.
- Abnormal T wave morphology including notching and low amplitude.
- *Torsade de pointes* seen during events.

#### Acute management

- For *torsades de pointes*, perform emergent defibrillation followed by administration of magnesium sulfate and possibly lidocaine.
- Correct underlying problem if acquired long QT.
- Intravenous beta-blockade may calm an adrenergic storm.

#### Further work-up and management in suspected congenital long QT syndrome

- Obtain thorough family history of rhythm abnormalities, sudden death, deafness.
- Ascertain all medications.
- Review history of event that may have triggered arrhythmia.
- Obtain electrolytes and treat underlying abnormalities.
- If presented with symptoms or documented VT, admit for observation, cardiology consultation and treatment.
- For patients presenting with non-cardiac issues and noted to have abnormal QTc interval, out-patient cardiology follow-up may be arranged.
- Restrict all strenuous activity pending cardiology follow-up.
- Provide list to the patients of medications known to prolong QT that should be avoided.
- Immediate family members should also be screened with 12-lead EKGs.

#### Prognosis

Prognosis is poor in untreated symptomatic patients, with an annual mortality of 20%. B-blockers are the mainstream therapy and reduce risk of sudden death to about 6% annually but do not eliminate it completely.[12] High risk patients may benefit from ICD placement which has been shown to reduce mortality risk. Factors known to increase risk include history of previous syncope, deafness, previous torsade, female gender and genotype.[13]

## CONGENITAL HEART DISEASE

Patients with congenital heart disease are at lifelong risk for the development of arrhythmias. Types of arrhythmias depend on the underlying cardiac anomaly and more importantly on the surgical repair, and can range from atrial flutter to ventricular fibrillation. (See Table 1) ED management should focus on understanding the cardiac anatomy and previous surgical interventions and to focus on how these can predispose patient to certain rhythm disturbances. In the following section, we describe some of the most common congenital heart diseases and their associated arrhythmias.

**Table 1:** Congenital heart disease and associated arrhythmias

Congenital heart disease	
Disease	Associated arrhythmia
Tetralogy of Fallot	Atrial tachycardia
Double outlet right ventricle	Ventricular tachycardia
	Sinus node dysfunction
Transposition of the great arteries	Ventricular arrhythmias
	Atrioventricular block
Ebstein's anomaly	Supraventricular tachycardia
Ventricular septal defect repair	Heart block
	Ventricular arrhythmias
Atrialseptal defect	Atrial tachycardia
Atrial septal defect repair	Sinus node dysfunction

### 1. Ventricular septal defect repair

Ventricular septal defects (VSD), by far the most common congenital heart defect, are not generally associated with rhythm abnormalities. In the present era, clinically significant VSDs are repaired in early childhood, decreasing the incidence of arrhythmias. By avoiding right ventriculotomies, newer surgical approaches have significantly altered the incidence of ventricular arrhythmias. Operated patients, however, remain at an increased risk for developing heart block, even late after repair. The presence of a small, hemodynamically insignificant VSD does not increase the risk of arrhythmia.

### 2. Atrial septal defect repair

Atrial septal defect (ASD) occurs in 5–10% of all congenital heart defects and is found in 30–50% of children with other types of congenital heart disease as well.[14] In large defects, atrial tachycardias can develop secondary to chronic volume overload and atrial dilation. Early repair has been credited for improved survival and decreased incidence of late arrhythmias. Possible surgical complications include injury of the SA node producing sinus nodal dysfunction and secondary bradycardia or tachy-brady syndrome.

### 3. Tetralogy of Fallot

Tetralogy of Fallot (TOF) is the most common cyanotic heart disease, representing 3.5–9% of patients with CHD.[15] Arrhythmias, both atrial and ventricular, occur in unrepaired and repaired patients [Figure 7] A meta-analysis study by Walsh *et al*. found an incidence of 2–6% of arrest or sudden late death in patients repaired during infancy.[16] Although atrial tachycardias are actually more common, the primary concern in TOF is VT, with a long-term mortality risk estimated at 3–6%.[17] Sinus node dysfunction and atrial reentry tachycardias have also been described, with an incidence of 20–30%.[18] A large multicenter trial by Gatzoulis *et al*, assessing risk factors for VT and sudden death in TOF patients found that QRS duration of >180ms, an increasing QRS duration, older age of repair, and/or presence of right ventricular patch were associated with increased risk of arrhythmia.

### 4. Transposition of the great arteries

The so-called “congenitally corrected” or L-transposition of the great arteries is rare, estimated to occur in 1% of all patients with congenital heart disease, but risk of arrhythmia is high. In this condition, the right atrium is attached to the left ventricle which empties to the pulmonary artery, and the left atrium enters the right ventricle and thus into the aorta. The incidence of spontaneous complete heart block has been reported to be as high as 22% owing to intrinsic abnormalities of the His bundle.[19]

D-transposition of the great arteries (right ventricular connection to aorta and left ventricular connection to pulmonary artery) is much more common, and was a uniformly lethal disease prior to corrective surgery. With current surgical techniques, arrhythmia risk is relatively low. During outpatient follow-up, Rhodes and colleagues found an incidence of 4.4% of some form of AV block. SVT was seen in 5%, whereas almost half of the patients showed evidence of ventricular arrhythmias, mostly isolated premature beats, 6–12 months following repair.[11]

### 5. Ebstein’s anomaly

Ebstein’s anomaly is a very rare condition in which the tricuspid valve leaflets have an abnormal attachment causing displacement of the valve into the right ventricle, so called “atrialized” ventricle. Patients are at increased risk for the development of SVT, with an incidence as high as 42%.[20] In the setting of severe tricuspid regurgitation and/or atrial septal defects, tachycardias may be poorly tolerated. Patients with Ebstein’s anomaly are also at increased risk of atrial flutter and fibrillation due to the enlarged right atrium.

## CARDIOMYOPATHIES

Cardiomyopathies are a group of disorders of intrinsic muscle disease. These patients are at increased risk for atrial and VTs. Treatment can be challenging and ICD implant is being used increasingly in these populations.

### 1. Hypertrophic cardiomyopathy

Patients with hypertrophic cardiomyopathy (HCM) are at increased risk of malignant VT and sudden death. Multiple risk factors have been implicated, including family history of sudden death, history of syncope, dramatic septal thickness, non-sustained VT and inappropriate blood pressure response to exercise. Death is often sudden and usually occurs in the setting of vigorous exertion with an annual incidence of 3–5% in children and adolescents with HCM.[21] Current therapy includes exercise restriction and the use of beta-blockers or calcium channel blockers for symptomatic patients. Surgery is recommended for drug refractory cases with marked left ventricular outflow tract obstruction and ICD implantation for primary prevention in high-risk patients and secondary prevention in patients with syncope or documented VT.

### 2. Dilated cardiomyopathy

Dilated cardiomyopathy (DCM) is the most common severe cardiomyopathy, accounting for more than 50% of pediatric cases.[8] Etiology of DCM is broad and includes infectious, immunologic, familial, metabolic, toxic and arrhythmic causes. Although these patients are at risk for VT, SVTs are more common. It is sometime difficult to distinguish atrial tachycardia from sinus tachycardia, but in general, average rates exceeding 150 bpm highly correlate with atrial tachycardia. Treatment is targeted at symptoms, ventricular dysfunction and underlying rhythm disturbance. Prophylactic ICD use in the pediatric population is not as clear as in adults with ischemic cardiomyopathies.

**Figure 8:**
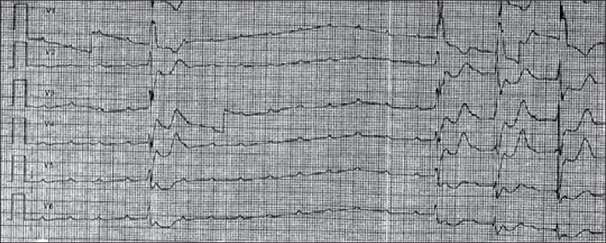
Atrioventricular block in patient with myocarditis. Required temporary pacing for 3 days with resolution of heart block

Myocarditis, a common precursor to DCM, may present with sudden death. Although the exact incidence of arrhythmias associated with myocarditis is unknown, VTs and high-degree AV block are not uncommon [Figure 8].

### 3. Arrythmogenic right ventricular dysplasia

Arrythmogenic right ventricular dysplasia (ARVD) is a genetic myopathy involving fibrofatty degeneration of the right ventricle and is commonly associated with VT and sudden death. Inheritance is mainly autosomal dominant but sporadic cases have been described. Peak age of presentation is late teenage years to the 20s, and a common presenting symptom is syncope with exercise. Electrocardiographic findings include right ventricular conduction delay, inverted T waves in the right precordial leads inappropriate for age and PVCs of right ventricular morphology. Treatment usually involves ICD implantation.

## BRADYCARDIAS

Bradycardias are a group of disorders that include sinus node dysfunction and abnormal conduction through the AV node. AV conduction abnormalities include first-, second- or third-degree heart block. First-degree heart block, prolongation of PR interval without loss of conduction, does not cause bradycardia or hemodynamic instability and is primarily of interest as a marker for an underlying cause. Causes of first-degree block are numerous and include enhanced vagal tone, previous cardiac surgeries, myopathies, and infections including Lyme disease, myocarditis, endocarditis and hypothyroidism.

### 1. Sinus bradycardia and sinus node dysfunction

Sinus bradycardia can result from multiple disease states, most of which are not primary cardiac. The exception is sinus node dysfunction, an abnormality of impulse generation and propagation of the sinus node usually caused in pediatrics by direct injury or disruption of blood supply to the node from previous cardiac surgery. Associated atrial reentry tachycardias are common and when they occur, the term “brady-tachy syndrome” is applied. Etiology of sinus bradycardia other than congenital heart disease is diverse and includes increased intracranial pressure, electrolyte abnormalities, respiratory compromise, hypothyroidism and certain medications. The need for acute treatment for sinus bradycardia itself is rare. The presence of sinus bradycardia in an otherwise healthy appearing child is generally of no concern, although anorexia nervosa should be considered. In the moribund patient with sinus bradycardia, urgent determination of the underlying cause is of paramount importance. Atropine and epinephrine increases sinus rates in most patients. Symptomatic patients with true sinus node dysfunction and/or those with coexistent tachycardias are likely to require elective pacemaker implantation.

### 2. Second-degree heart block

Second-degree heart block refers to intermittent failure of conduction through the AV node. It is further subdivided into Mobitz type I (Wenkebach), a gradual PR interval prolongation followed by a nonconducted beat, and Mobitz type II, an abrupt loss of conduction without previous change in PR interval duration. Mobitz type I is generally more benign and may be a normal finding in adolescents during sleep. Other causes are similar to those described for first-degree heart block. Treatment is aimed at underlying reversible causes. Progression to higher degree block has been reported and symptomatic patients may benefit from treatment with atropine or isoproterenol. Mobitz type II is thought to be more likely to progress to complete heart block. Elective pacemaker implantation has been advised for symptomatic patients in this group.[22]

### 3. Third-degree AV block

Third degree AV block is the absence of conduction from the atria to the ventricle, manifested by AV dissociation. QRS duration of the escape rhythm may be normal or prolonged. Third-degree block can be congenital, mainly associated with maternal collagen vascular disease or congenital heart disease, or acquired, due to AV node injury from cardiac surgery, viral myocarditis, Lyme disease, or metabolic or neuromuscular disorders. Many children are asymptomatic when diagnosed. In infancy, symptomatic patients may appear severely ill at presentation. Older children and adolescents may present with symptoms of exercise intolerance, fatigue, dizziness, syncope or signs of congestive heart failure. Sudden death may occur. Most patients eventually require pacemaker implantation, unless the cause is reversible such as in Lyme disease, where temporary pacing may suffice.

## CONCLUSION

Proper diagnosis and management of the diverse rhythm disturbances in children is challenging. Emergency healthcare providers must be comfortable in performing a systematic evaluation and interpretation of ECG findings. An understanding of the variety of diseases that can predispose children with normal hearts or with structural heart disease to arrhythmias is essential for appropriate treatment.
